# Mouse pulmonary response following solid surface composite dust inhalation

**DOI:** 10.1080/08958378.2024.2447699

**Published:** 2025-01-22

**Authors:** W. Kyle Mandler, Walter G. McKinney, Mark Jackson, Alycia K. Knepp, Sarah L. Keeley, Sherri A. Friend, Lori A. Battelli, Yong Qian

**Affiliations:** Health Effects Laboratory Division, National Institute for Occupational Safety and Health, Morgantown, WV, USA

**Keywords:** Solid surface composite, particle, inhalation, alumina trihydrate, methyl methacrylate

## Abstract

**Purpose:**

Pulmonary exposure to emissions from manipulating solid surface composite (SSC) materials has been associated with adverse health effects in humans and laboratory animals. Previous *in vitro* and *in vivo* investigations of SSC toxicity have been limited by particle delivery methods that do not fully recapitulate the workplace environment. This study sought to determine the acute SSC-induced pulmonary responses *via* whole-body inhalation exposure.

**Materials and Methods:**

A chamber for dust particle generation and an exposure system for characterization and animal exposures was constructed. The system successfully generated SSC at a concentration of 19.9 ± 1.5 mg/m^3^. The aerosol count median aerodynamic diameter was 820 nm. First, C57BL/6 mice were exposed to SSC particles for 4 h (*n* = 6) or filtered air control followed by euthanasia either immediately or 24 h post-exposure. Lungs were analyzed for aluminum (Al) content using inductively coupled plasma atomic emission spectroscopy (ICP-AES) which measured a lung deposition of 19.13 ± 5.03 µg/g elemental Al, or approximately 64 µg/g SSC dust. Second, a group of mice (*n* = 9) was exposed to SSC particles at 20 mg/m^3^ for 4 days, 4 h/day to assess the acute and sub-chronic pulmonary effects of SSC inhalation. Animals were euthanized at 1- and 56-days post-exposure.

**Results:**

Total estimated pulmonary deposition for these animals was 49.2 µg SSC dust/animal. No histopathologic changes were observed at any post-exposure time point; however, BALF total protein was increased at 1-day post-exposure.

**Conclusions:**

We conclude that exposure to dust from cutting SSC at this dose and post-exposure durations induces mild, transient inflammation.

## Background

Solid surface composites (SSC) are a category of synthetic, non-porous, and relatively hard material commonly used in the construction of countertops, workshop benches, furniture, and architectural cladding. While the exact formulations are proprietary and may vary slightly between manufacturers, SSC is composed of a mixture of about 70% alumina trihydrate (Al(OH)_3_, ATH) bound in an acrylic polymer matrix (DuPont 2018). Trace amounts of pigments, metals, and hardeners may also be present depending on the application (DuPont 2018). SSC are typically extruded into sheets or slabs in a factory, then transported to local installers where they are cut, drilled, ground, and polished into the required dimensions for each project. These processes generate respirable airborne particulates and volatile organic chemicals (VOCs) (Qi et al. 2016). Substantial respirable dust exposure has been observed in an SSC fabrication facility (Vinnikov et al. 2021). Based on 29 full working-day samples for three workers during ordinary working conditions, the median respirable PM value was 2.0 mg/m^3^, with a peak exposure during most workdays over 10 mg/m^3^, ranging as high as 25.4 mg/m^3^. The average value observed (Vinnikov et al. 2021) was below the OSHA permissible exposure limit for respirable particles not otherwise regulated (PEL, PNOR) 8-h time-weighted (TWA) average of 5 mg/m^3^. Two cases have been described for pulmonary SSC dust exposure-induced idiopathic pulmonary fibrosis (Corwin et al. 2024; Raghu et al. 2014).

The components of SSC, including ATH, and methacrylate have been associated with lung disease both in the occupational and laboratory setting. Specifically, Shaver and Riddell reported a high prevalence (approximately 20%) of rapidly fatal pneumoconiosis among alumina abrasives manufacturing workers (Shaver and Riddell 1947). Similar findings have also been reported in workers exposed to fine alumina dust used in pyrotechnic manufacture (Mitchell et al. 1961; Gaffuri et al. 1985). Additionally, the production of plastic-containing acrylate polymers, found within SSC emissions, generates ultrafine particles and volatile organic compounds (VOCs) (Unwin et al. 2013). Notably, prolonged pulmonary exposure (5–13 months) to polyacrylate nanoparticles in a group of female workers led to shortness of breath, pleural effusions, and nonspecific inflammatory, fibrotic, and granulomatous lung pathology (Song et al. 2009). Furthermore, transmission electron microscopy confirmed the presence of acrylate nanoparticles within the cytoplasm of pulmonary epithelial and mesothelial cells. Animal studies further corroborate these concerns. Intratracheal instillation (IT) of ATH in rats resulted in dose-dependent fibrotic lung lesions. At 70 mg of ATH, numerous confluent nodules were observed after 60 days, progressing to firm fibrotic masses at 180 mg/rat. By day 210, the majority of lung tissue had transformed into a mass of collagenous tissue (Stacy et al. 1959). Inhalation exposure to Al oxide nanoparticles for 28 days in male rats elicited dose-dependent lung inflammation and potential respiratory distress (Kim et al. 2018). In an experiment simulating Al refinery worker exposure, mice exposed for 1 hr to 8 mg/m^3^Al refinery dust (ALUM) displayed significantly impaired lung mechanics and inflammatory responses compared to controls (Mazzoli-Rocha et al. 2010). While direct data on workplace exposure to SSC emissions is limited, these findings collectively suggest that constituents of these aerosols possess the potential to induce lung injury in humans.

Our group has investigated the pulmonary toxicity of SSC particulate exposure using *in vitro* and *in vivo* methods. Using transformed THP-1 cells as a model of alveolar macrophages, we observed that viability was reduced by 15% and 19% after 24 h exposure to 5 and 10 µg/well SSC particles, respectively. Supernatant lactate dehydrogenase (LDH) activity was increased by 40% and 70% when compared to control. Reactive oxygen species (ROS) production and inflammatory cytokines also increased in a dose-dependent manner(Mandler et al. 2020). Aspiration of varying doses of SSC in mice (62.5 µg–1000 µg) resulted in dose-dependent lung inflammation. Bronchoalveolar lavage fluid (BALF) analysis revealed elevated lactate dehydrogenase (LDH) activity, inflammatory cells, and pro-inflammatory cytokines at higher doses. Histopathological examination at day 1 displayed acute alveolitis across all exposure groups, largely resolving by day 14. Notably, all groups exhibited persistent alveolar particle deposition and granulomatous mass formation at day 14. Additionally, Picrosirius red staining of lung sections in the 1000 µg group indicated increased collagen deposition, suggesting potential fibrotic development (Mandler et al. 2019). Considering these findings, we concluded that pulmonary exposure to SSC particles, especially at higher deposition levels, could induce lung inflammation and fibrogenesis. However, these studies were limited in that the particles delivered to the animals and cells were not necessarily representative of aerosols encountered by workers.

Previous*in vitro* and *in vivo* investigations of the toxicity of these materials all relied on particles generated, collected, and stored prior to exposure. While those studies provided valuable information identifying the possible hazard inherent in these materials, those exposure models did not entirely recapitulate the workplace environment. Most particles used for these studies were fine or ultrafine (<2.5 µm), however, a portion of them were quite large (>5 µm geometric diameter) and would thus be less likely to deposit in the alveoli than smaller inhaled aerosols (Mandler et al. 2019, 2020). Additionally, particle aging has been shown to alter the toxic effects of both organic (Künzi et al. 2015; Wei et al. 2020) and inorganic (Smith et al. 2018; Dong et al. 2020) particles, both in a positive and negative sense. Furthermore, SSC sawing produces methyl methacrylate and butyl acrylate volatile organic compounds(VOCs) (Kang et al. 2019), which would not be captured in the particles collected and stored for later use but are associated with cases of occupational interstitial lung disease (Piirila et al. 1998, 2002; Aydin et al. 2002; Kim et al. 2013). Considering the mixed nature of SSC, without a real-time generated SSC emission paradigm, it would not be possible to have a comprehensive assessment of SSC toxicity. With this obstacle in mind, our group developed an apparatus which can generate SSC sawing particles in real-time and simultaneously deliver them to rodents *via* whole body inhalation in a highly reproducible manner. The objective of the present study was to characterize the acute pulmonary toxicity of 4 days of inhalation exposure to SSC aerosols generated in real-time at 1-, and 56-days post-exposure.

## Materials and methods

### Automated countertop cutting exposure system design

**Figure 1.**
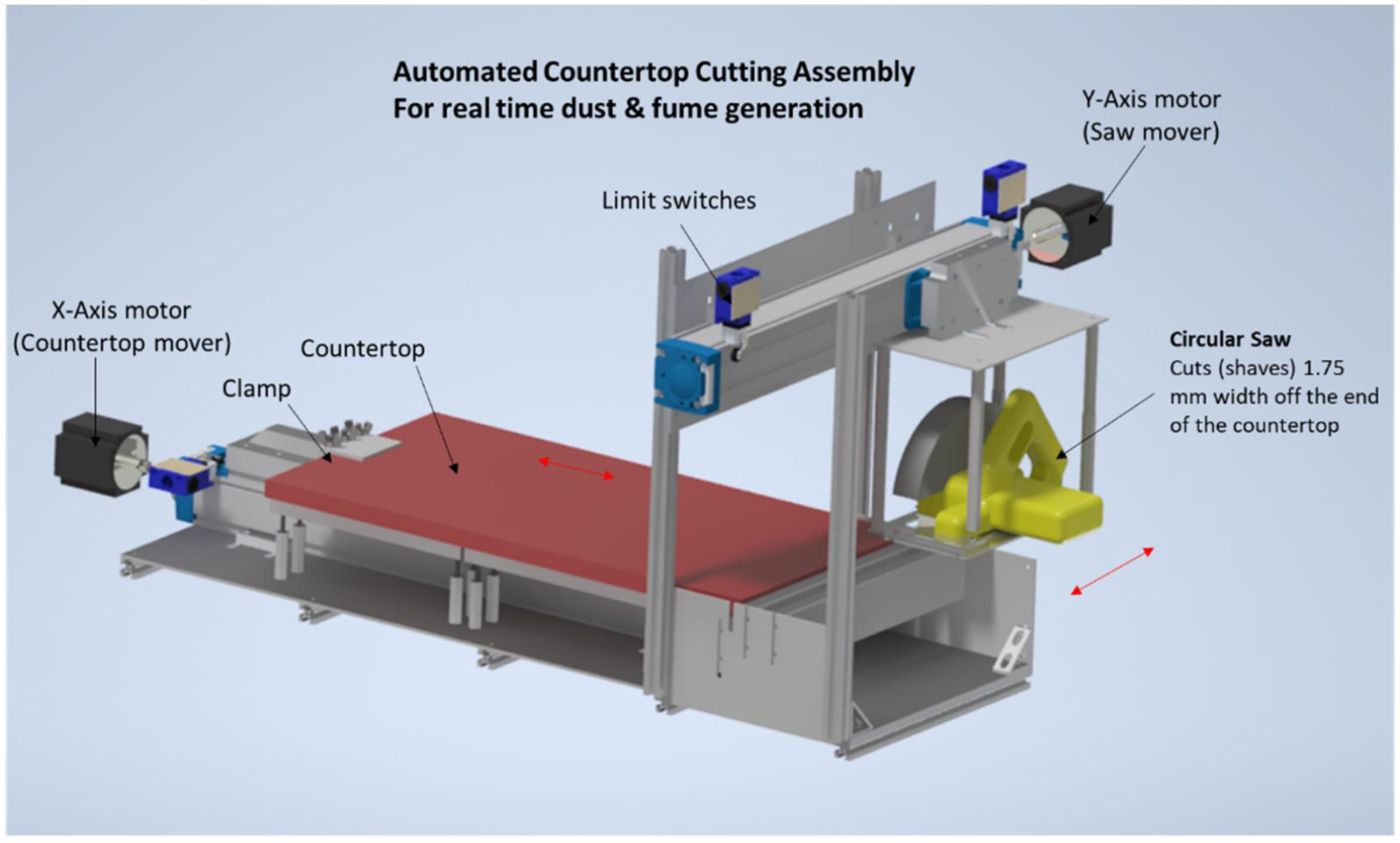
Automated countertop cutting assembly for real time dust generation.

**Figure 2.**
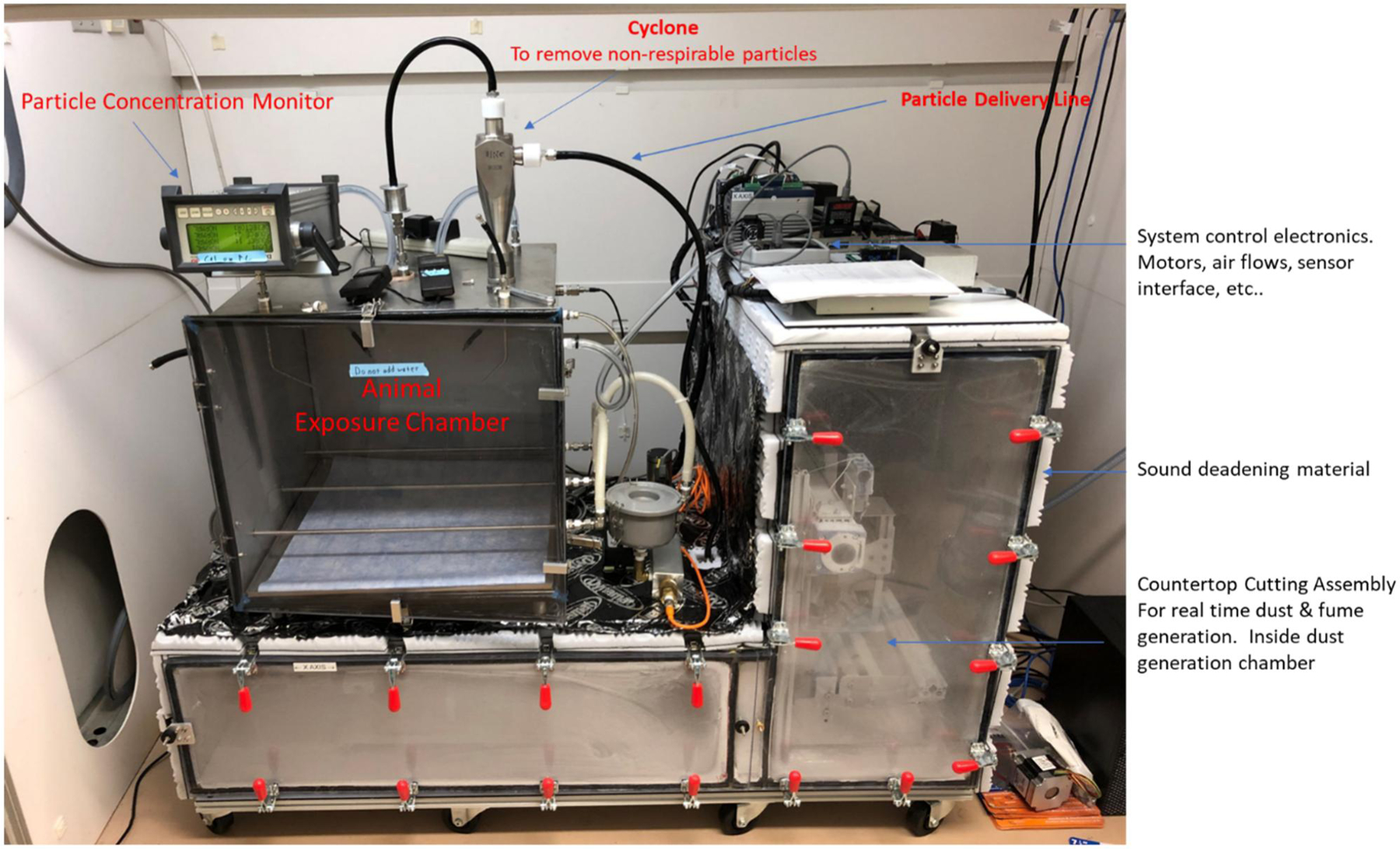
Picture of automated countertop cutting inhalation exposure system with labeled components.

A simplified diagram of the automated countertop cutting assembly is depicted in Figure 1, and a photograph with components labeled the automated countertop cutting inhalation exposure system is presented in Figure 2. Custom exposure system software automatically controlled chamber air flows, particle concentration, and exposure duration. The software also monitored exposure chamber temperature, relative humidity, and CO_2_ levels. During exposures, the exposure chamber exhaust airflow was held constant at 30 L/min. This level of airflow was sufficient to maintain CO_2_ levels below 2,500 PPM as recommended by NIOSH Morgantown’s internal animal and care use committee. Air entered the top of the exposure chamber either from being drawn through an airtight chamber that housed the automated saw assembly or from a mass flow controller (MCR-50, Alicat Scientific, Tucson AZ) that provided filtered air. Typically, the dilution air flow was set to zero by the exposure system software during exposures until the target exposure time was reached. Immediately before the air entered the exposure chamber it traveled through a cyclone (Model 2000, URG, Chapel Hill, NC), to remove particles with aerodynamic diameters larger than 6 µm.

**Figure 3.**
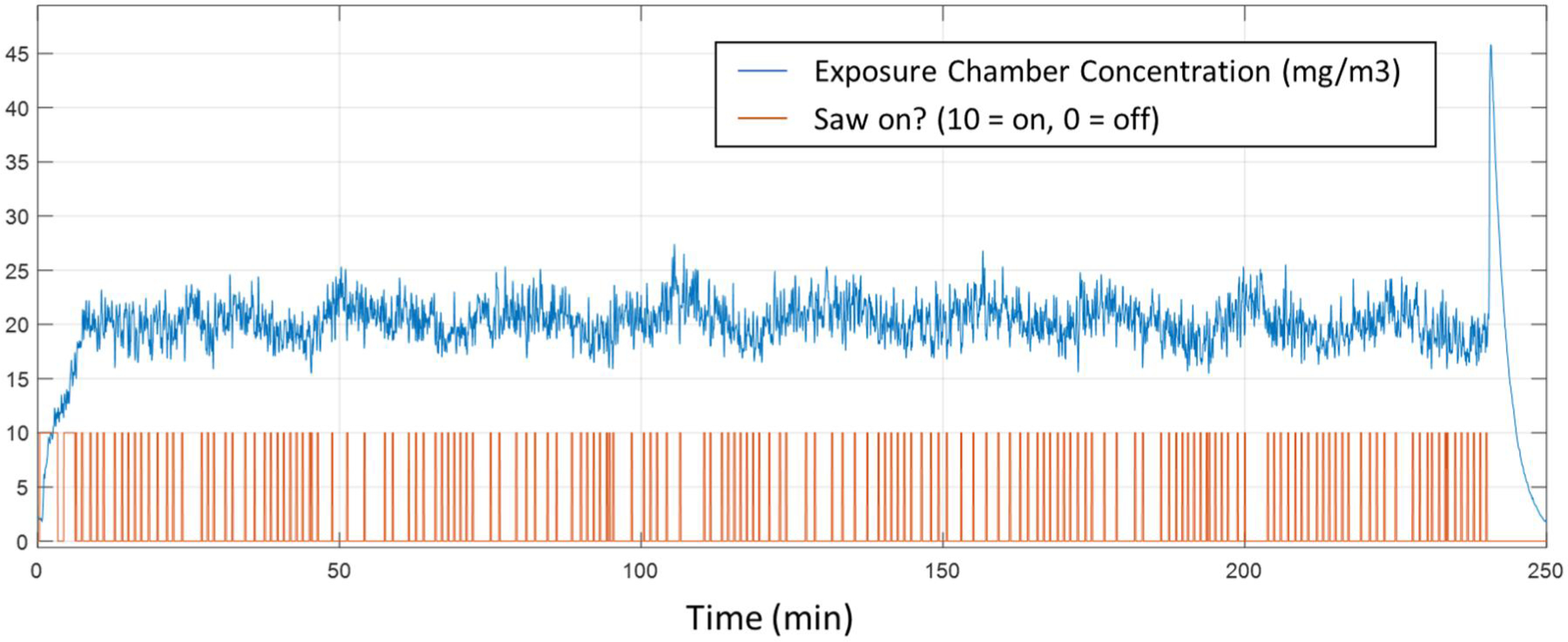
Representative inhalation exposure chamber concentration vs. time during an animal exposure.

The aerosol mass concentration inside the exposure chamber was continuously monitored with a Data RAM (DR-40000 Thermo Electron Co.) and gravimetric determinations (37 mm cassettes with 0.45 µm pore-size Teflon filters, (1 L/min sample flow)) were used to calibrate and verify the Data RAM readings during each exposure run. For this study the target concentration was a constant 20 mg/m^3^ over 4-h long exposures. Single day and four consecutive day exposures were conducted. The custom software automatically determined when to saw and when to stop based on exposure chamber concentration readings. The parameters of this algorithm (S1), length of cut, and error limits were optimized by trial and error over several test exposure runs with an empty exposure chamber. Figure 3 shows a plot of the exposure chamber concentration versus time during one of the inhalation exposure runs. The short duration spike at the end of the exposure was from the dilution air being automatically set to maximum flow. This change in flow would often cause particles to break loose from tubing walls. Even with this spike, using the dilution air cut the time required to reduce the concentration at the end of an exposure run.

Exposure chamber pressure was monitored using a differential pressure transducer (Model 264, Setra Systems, Inc. Boxborough MA). Under typical exposure conditions dilution air was set to zero and the pressure inside the exposure chamber was −6.35 cm-H_2_O to ambient. This slight negative pressure would pull air from the saw assembly housing chamber into the exposure chamber *via* conductive silicone tubing (78.7-mm ID). The temperature and relative humidity inside the exposure chamber were also continuously monitored by using an electronic probe (HMP60, Vaisala Corporation, Helsinki Finland).

### Automated countertop cutting assembly

A 3D rendering of the automated cutting assembly is shown in Figure 1. Two linear actuators were used (Movopart M75 model, Thomson Linear motion system), driven with stepper motors (Powerpak K3 series, Kollmorgen). A pair of Kollmorgen P6000 stepper drivers were used to interface the custom exposure software with the stepper motors. The first motor (X-axis) moved the countertop away from or in front of the circular saw. The second motor (Y-axis) moved the circular saw to cut through the countertop. Limit switches were used to detect home positions and ensure a safe range of motion. The circular saw (Model number DWE575SB, DeWALT, Towson MA) used anOshlun (SBNF-072560) 18.42 cm diameter 60 teeth, aluminum cutting, professional grade C-1 carbide tip, triple chip grind with 1.59 cm arbor blade. This blade is recommended for cutting solid surface countertops. A 1.75 mm width, the approximate width of the blade, was shaved off the end of the countertop during each cut. This was done to avoid producing large solid sections of cutoff material that could obstruct subsequent cutting. The type of countertop used in our experiments was Dupont Corian light gray in color and measured 76.2-by-38.1-by-1.27 cm.

The automated cutting assembly was housed in a custom airtight 16-gauge stainless steel chamber with a removable clear polycarbonate front door as shown in Figure 2. A silicon gasket and several latches ensured an airtight seal between the door and the chamber. Sound-deadening material was affixed to the outside of the chamber to reduce the noise generated by cutting. Air entered the saw assembly housing chamber on the far-left side by first passing through a HEPA filter. Air with countertop particles exited the cutting assembly chamber from a port located on top of the chamber approximately 0.3 m from the cutting blade. The saw housing chamber also acted as a cart body that held the exposure chamber, mass flow controllers, and all interface electronics. The entire system was on wheels and designed to fit inside an 8-foot walk-in fume hood.

### Inhalation exposure chamber

An airtight 55.9 by 55.9 by 50.8-cm (L × W × H) exposure chamber was constructed out of 16-gauge stainless steel with a clear polycarbonate door. A stainless-steel cage rack that could hold up to 36 mice in individual cage partitions was used to house the mice during exposures. The cage rack rested on top of cage support beams which were 0.95 cm outside diameter stainless steel tubes with small holes (3.3 mm diameter) drilled into the undersides. The tubes were connected to house vacuum through a mass flow controller (MCRW-50-DS, Alicat Scientific, Tucson AZ). Each hole was placed at the center of each cage partition such that aerosols would be drawn to each animal’s breathing space. Air entered from the top center of the chamber through a dispersion nozzle to aid aerosol mixing. This type of chamber, the Cube 150, was previously described by Goldsmith et al. (2011). The Cube 150 was previously tested for concentration homogeneity within the animal cage partitions using aerosol and was reported to have less than 5% variation between all animal locations within the chamber. The exposure chamber air flow rate was 30 L/min. Aerosol homogeneity in the exposure chamber is achieved through several methods. First, the inlet nozzle is designed to disperse aerosol evenly throughout the chamber, second, exhaust ports placed below each individual animal cage draw air evenly through the breathing space of each animal. Additionally, during exposures that span multiple days, animal location within the chamber is randomized each day.

### Particle characterization

Aerodynamic particle size distribution (count based) data were collected (APS Model 3321, TSI Inc., Shoreview, MN) while cutting at several different saw travel speeds. The samples were taken inside the inhalation exposure chamber a few cm above the animal cages, and exposure chamber concentration was held constant at 20 mg/m^3^ during the sample collection. Each sample lasted 5 min. The sample flow rate for the APS was 5 L/min, and its detectable size range is from 0.5 to 20 µm. This data were collected to determine if travel saw speed had a significant effect on the respirable particle size distribution. A second aerodynamic particle sizing instrument with a smaller size detection range (0.07 to 10 µm), but a smaller total number of size bins, was also used to collect data (ELPI Model ELPI classic, Dekati Ltd., Finland). A 5-min sample was taken from inside the exposure chamber with the ELPI at a cutting speed of 2.5 mm/sec. The ELPI’s sample flow was 10 L/min. Mass-based aerodynamic particle size distribution was determined in the exposure chamber by using a Micro-Orifice Uniform Deposit Impactor (MOUDI, MSP Model 110 R, MSP Corporation, Shoreview, MN). Cutting speed for this sample was 2.5 mm/sec, and its sample flow was 10 L/min. The countertop cutting particles inside the exposure chamber were sampled at 1 L/min onto polycarbonate filters (Whatman, Clinton, PA) and imaged with a scanning electron microscope (FE-SEM; Hitachi, S-4800, Tokyo, Japan). All of these sizing samples were taken without animals inside the chamber while the system ran in the same manner as an exposure run.

### Animals

Male C57BL/6J mice (4 weeks old, 20–25 g) were purchased from The Jackson Laboratory (Bar Harbor, ME), housed in ventilated polycarbonate cages, and acclimated for at least 7 days before the study began. The animals were provided HEPA-filtered air, irradiated Teklad 2918 diet (Harlan, Madison WI), a combination of ALPHA-dri and Teklad sani-chips as bedding, and tap water ad libitum. Mice were housed in two per cage with a 12-h light-dark cycle, and the facility was maintained at 22 ± 2 °C and 40–60% humidity. The study protocol was reviewed and approved by the CDC-Morgantown Institutional Animal Care and Use Committee. The CDC-Morgantown Institutional Animal Care and Use Committee is accredited by AAALAC International.

### Experimental design

Two separate exposures were conducted for this study. In the first exposure, following acclimatization, the animals (*n* = 6) were exposed by whole-body inhalation to HEPA- and carbon-filtered air in the exposure chamber with the saw running but not cutting, or an average concentration of 20 mg/m^3^ SSC-emissions, for 4 h. Immediately following exposure or 24 h-post, the mice were euthanized following an intraperitoneal injection of 100–300mg sodium pentobarbital/kg body weight (Fort Dodge Animal Health; Fort Dodge, IA). In the second exposure, the animals were randomly divided into two groups (*n* = 9 per group) and exposed by whole-body inhalation to HEPA- and carbon-filtered air in the exposure chamber with the saw running but not cutting, or an average concentration of 20 mg/m^3^SSC-emissions, for 4 h per day for 4 days. Animals from the second exposure were euthanized at 1- and 56-days post-exposure. Upon euthanasia following both exposures, bodyweight was measured and whole blood was collected *via* cardiac puncture and transferred to a vacutainer containing EDTA (Becton-Dickinson; Franklin Lakes, NJ) for whole blood hematological measurements. Each group was divided into subgroups, with the right lungs (*n* = 4)frozen for biochemical analysis and the left lung fixed for histopathology. Bronchoalveolar lavage was performed on the remainder (*n* = 5, BALF collection further described below).

### ICP-AES Al analysis

Tissue Al concentrations were determined by Galbraith Laboratories, Inc. (Knoxville, TN), using inductively coupled plasma (ICPor ICP–atomic emission spectrometry (AES)). Briefly, duplicate samples of 150–300 mg of lung tissues were digested in concentrated nitric acid with the addition of hydrogen peroxide and heating in a digestion block and analyzed using ICP–AES (Galbraith method ME-70) using a PerkinElmer Optima 4300 (PerkinElmer, Waltham, MA). The instrument quantitation limit for the analysis was 0.0154 mg/L.

### Bronchoalveolar lavage

Bronchoalveolar lavage of the whole lung was performed by inserting a cannula into the trachea and gently flushing with Ca^2+^ and Mg^2+^ free phosphate-buffered saline (PBS), pH 7.4. The initial whole lung lavage was performed twice using 0.6 mL PBS for a collection volume of 1.2 mL, which was processed for cytokine and LDH activity analysis. Three subsequent lavages were performed using a 1 mL aliquot per lavage for a collection volume of 3 mL, which was utilized for cell differential quantification. BALF cells were collected through centrifugation (1,500 × g, 5 min, 4 °C) of the first and subsequent lavages, cell pellets were combined, washed with 1 mL PBS, and centrifuged (1,500 × g, 5 min, 4 °C). BALF cells were re-suspended with 250 µL PBS and total cell counts were determined using a Coulter Counter (Multisizer 4, Beckman Coulter, Brea CA). Cytospins of the BALF cells were generated using a Shandon Cytospin 4 (Thermo Fisher; Waltham, MA) and stained with modified Wright stain (Thermo Fisher). Cytospins were analyzed for cell differentials using an Olympus AX70 light microscope (Tokyo, Japan) coupled with Olympus DP73 camera, and analyzed using the Olympus CellSens Dimension program. Two-hundred cells per slide were counted.

### BALF LDH and total protein

Total protein and LDH activity were evaluated in the first acellular fraction of BALF. Analyses were conducted on the Synergy H1 Microplate Reader (BioTek; Winooski, VT) utilizing the Pierce BCA Protein Assay Kit (Fisher Scientific; Waltham, MA) for protein quantification and the Lactate Dehydrogenase Reagent Set (Pointe Scientific; Lincoln Park, MI) for LDH activity determination.

### BALF cytokine levels

The levels of pro- and anti-inflammatory cytokines in lung BALF were measured using a V-PLEX Pro-inflammatory Mouse panel(MSD; Meso Scale Discovery, Rockville, MD) according to the manufacturer protocol. IL1-β, IL-2, IL-4, IL-5, IL-6, IL-10, IL-12p7- tumor necrosis factor alpha, (TNFα), interferon (IFN)-γ, and neutrophil-activating protein 3 (KC/GRO), were measured from undiluted BALF. Data were acquired using a QuickPlex SQ 120 plate reader (MSD).

### Histopathology

Lung and nasal cavity were fixed in 10% neutral buffered formalin (NBF), embedded in paraffin, cut at 5 mm and stained with Hematoxylin and Eosin (H&E) for histopathological evaluation. Additional sections of lung were stained with Picrosirius Red. All glass slides were submitted to Experimental Pathology Laboratories (EPL), Inc., Durham, NC for examination by a board-certified pathologist using bright field and polarizing light microscopy (for visualizing particles). Findings were graded from one to five (1 = minimal; 2 = mild; 3 = moderate; 4 = marked; and 5 = severe), depending upon severity. A description of the scoring system can be found in S11.

### Blood processing and analysis

Whole blood collected from each mouse (*n* = 9/group)was transferred to a tube containing EDTA as an anticoagulant. Complete blood count (CBC) tests were performed post-exposure to evaluate hematological parameters, which included peripheral erythrocyte and leukocyte counts, leukocyte differentials (percent lymphocytes, neutrophils, monocytes, basophils, and eosinophils), platelet counts, mean platelet volume (MPV), hemoglobin levels, hematocrit, mean corpuscular hemoglobin (MCH) and hemoglobin concentration (MCHC), red blood cell distribution width (RDV), reticulocyte counts, mean platelet volume (MCV) using a ProCyte Dx Hematology Analyzer (IDEXX Laboratories, Inc., Westbrook, ME).

### Tissue oxidative stress markers

The right lung from each non-lavaged animal was homogenized with a Bead Mill 24 Homogenizer (Fisher Scientific International, Inc.; Hampton, NH) for 2 min at 4 °C in 1 mL cold PBS (pH 7.4) containing protease inhibitor cocktails and EDTA (Halt Protease Inhibitor Cocktails, Thermo Scientific, Waltham, MA). Data were acquired using a Synergy H1 Microplate Reader (BioTek, Winooski, VT). Tissue superoxide dismutase (SOD) activity was measured using ab65354 Superoxide Dismutase (SOD) Activity Assay Kit (Colorimetric) (Abcam, Cambridge, United Kingdom) and antioxidant capacity was measuring using Total Antioxidant Capacity (TAC) Assay Kit (ab65329). The values for each sample were normalized to sample total protein content determined using the Pierce BCA Protein Assay Kit (Fisher Scientific International, Inc., Hampton, NH).

### Statistics

Two-way ANOVA and subsequent *t*-tests on the main and interaction effects were performed. If one or more of the necessary ANOVA assumptions were violated, then a Kruskal Wallis test as an omnibus test and a Wilcoxon test on the main and interaction effects was used. For endpoints where some values fell below the assay lower limit of quantification (LLOQ), those values were replaced by the given LLOQ value divided by the square root of two.

## Results

### Particle characterization

**Figure 4.**
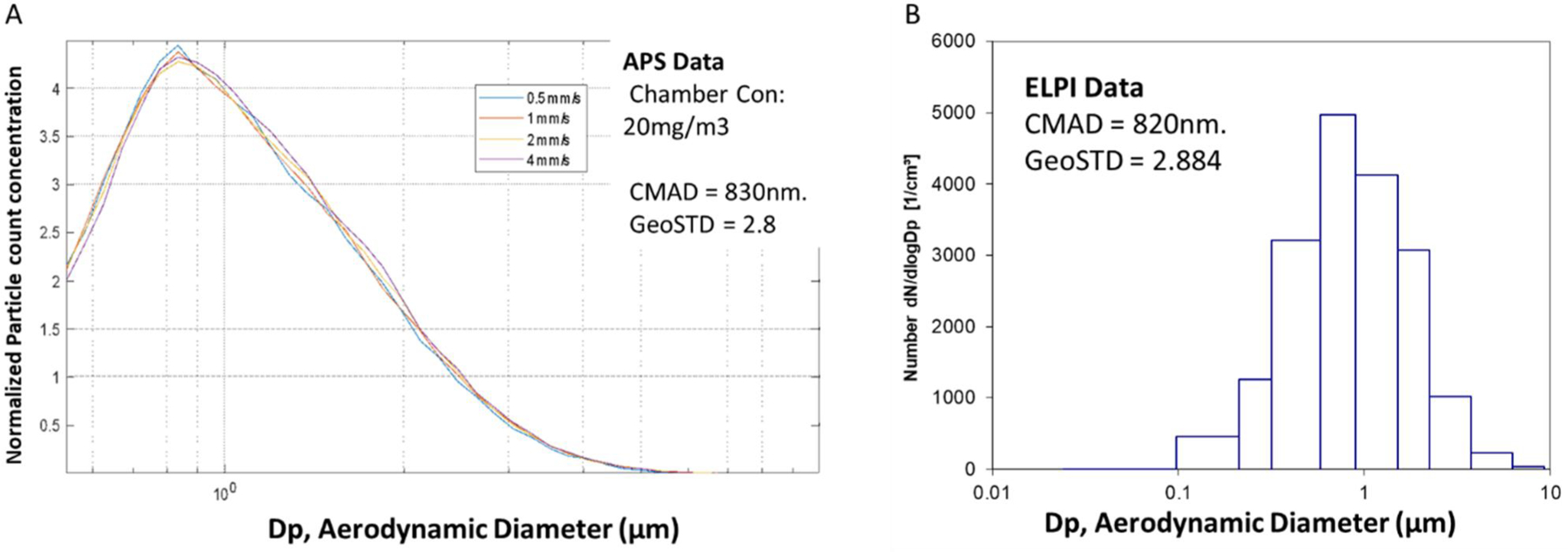
Particle size count-based distributions (APS data) at several cutting speeds (a), and ELPI data (B). Neither particle count concentration or CMAD varied appreciably between cutting speeds, and 2.5 mm/sec was selected for exposures.

The aerodynamic particle sizing data taken (APS Model 3321) from inside the exposure chamber, while cutting at several different saw travel speeds (0.5, 1, 2 and 4 mm/s), is shown in Figure 4(A). Saw travel speeds ranging from 0.5 mm/s to 4 mm/s did not have a substantial effect on the exposure chamber particle size. The count median aerodynamic diameter (CMAD) was 830 nm. The ELPI instrument was also used to collect particle size data inside the exposure chamber to determine if there was a second mode of particles less than 500 nm. The cutting speed during the ELPI data collection was 2.5 mm/s. These data are given in Figure 4(B). The ELPI software indicated a CMAD of 820 nm, with particles ranging in aerodynamic size from 0.1 to 5 µm.

**Figure 5.**
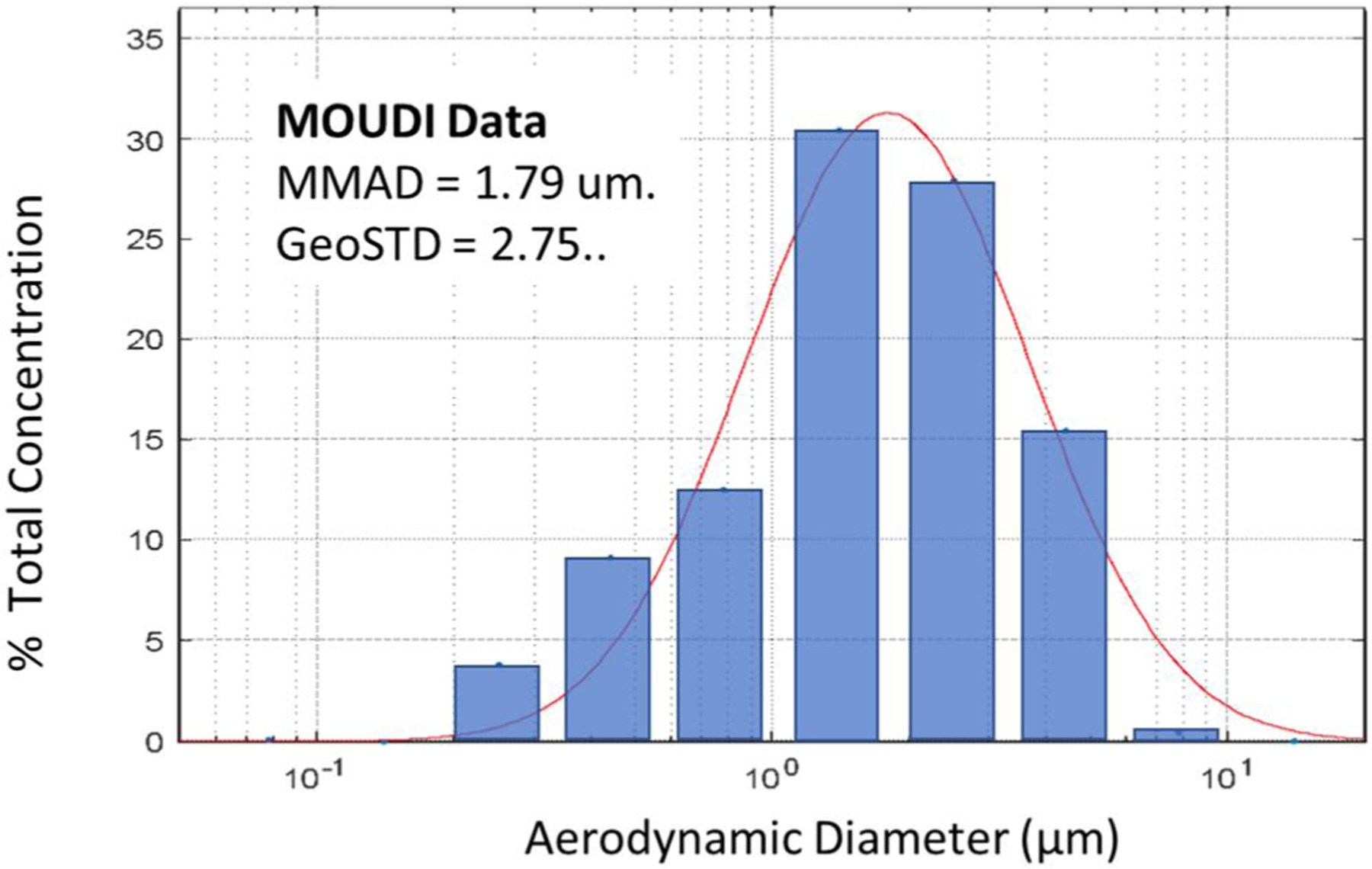
Particle size mass-based distribution (MOUDI data). The mass median aerodynamic diameter (MMAD) was 1.79 µm, with a geometric standard deviation of 2.75. Solid red line indicates fitted data fitted lognormal curve.

The APS and ELPI both provide count-based size distributions. A micro-orifice uniform deposit impactor (MOUDI) was used to determine the mass-based aerodynamic particle size distribution during typical exposure run conditions, 2.5 mm/s cutting speed and 20 mg/m^3^ concentration. These data are plotted in Figure 5. The mass median aerodynamic diameter was 1.78 µm with a geometric standard deviation of 2.75. The red line in the plot is a log-normal distribution curve fitted to the data.

**Figure 6.**
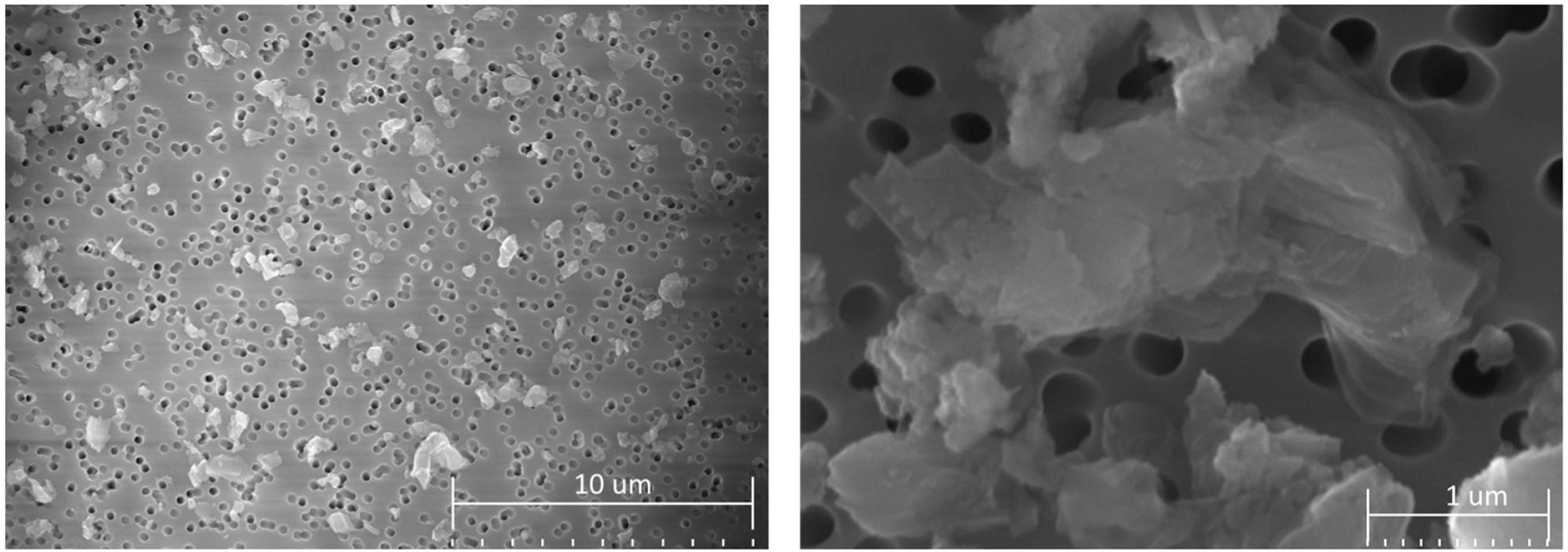
SEM images of particles sampled from the exposure chamber.

Scanning electron microscope (SEM) images of particles collected from the exposure chamber are shown in Figure 6. The small dark circles on the surface are pores in the filter material and the particles are deposited on top. The particles seem to have sharp edges and complex shapes, with rectangular being the most common. Physical particle sizes ranged from 0.2 to 4 µm in geometric diameter, with 1 to 2 µm being the most common. When single particles are inspected closely, they seem to be made up of thin sheet-like layers. SEM energy dispersive X-ray analysis (EDX) was used to verify the particles contained Al as expected (not presented).

### Exposure concentration

The aerosol mass concentration inside the exposure chamber was continuously monitored with a Data RAM (DR-40000 Thermo Electron Co.) and gravimetric determinations (37 mm cassettes with 0.45 µm pore-size Teflon filters, 1 L/min. sample flow) were used to calibrate and verify the Data RAM readings during each exposure run. For this study the target concentration was a steady 20 mg/m^3^ over 4-h long exposures. Single day and four consecutive day exposures were conducted. There were two separate single day exposures and the gravimetric data over these two days yielded an average concentration of 19.9 mg/m^3^ with a standard deviation of 1.5 mg/m^3^. For the 4-day exposures, the average daily concentration was 20.1 mg/m^3^ with a standard deviation of 1.2 mg/m^3^.

### Lung Al deposition and SSC lung burden

**Figure 7.**
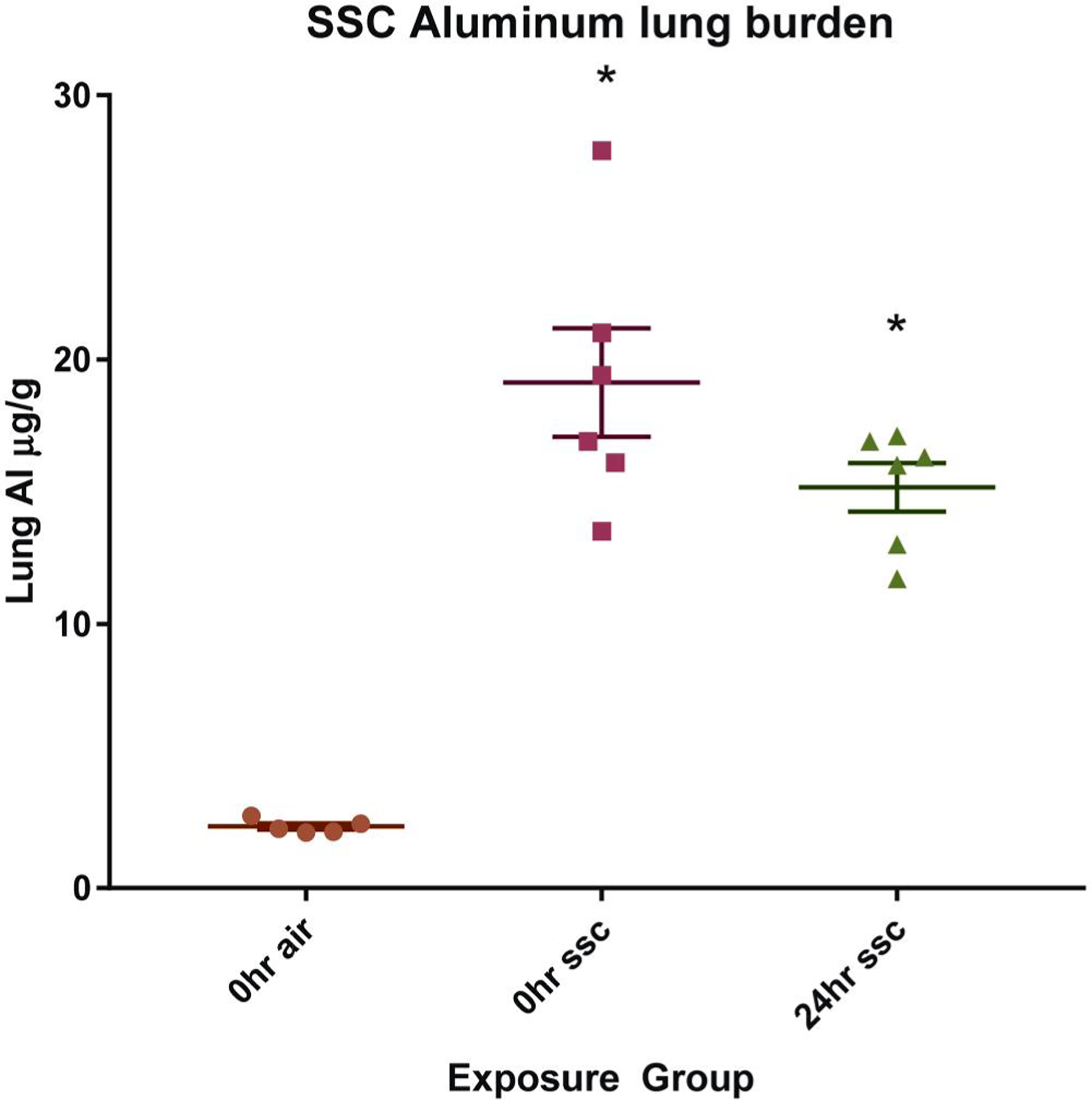
Lung aluminum content 0 and 24 h post-single, 4h SSC inhalation exposure (n 5–6). Lung aluminum, as measured using ICP-AES, was 19.13 ± 5.03 µg/g immediately following the exposure period. After 24 h, the concentration was 15.17 ± 2.26 mg/g; however, there was no statistical difference between this and the 0 h group. The air exposed control Al concentration was 2.33 ± 0.26 µg/g.

ICP-AES analysis of frozen whole lung tissue indicated that the mean Al concentration in the control animals was 2.33 ± 0.26 µg/g wet weight (Figure 7). At 0 h post-exposure the concentration was 19.13 ± 5.03 µg/g. At 24 h post-exposure, the Al concentration had decreased to 15.17 ± 2.26 µg/g, although this value was not significantly different from the 0-h concentration. Because elemental Al only comprises roughly 30% of the SSC composite (Qi et al. 2016), the actual lung burden can be estimated at 64 and 51 µg/g for the 0- and 24-h timepoints, respectively. The average lung weight for the 0 h exposure group was 205 mg, yielding an average of 13.2 µg of SSC lung deposition per animal, per 4-h day of exposure.

### Histopathology

H&E-stained tissue sections of left lung (LL) and nasal cavity (T1, T2, T3, and T4) were examined for routine histopathology using bright field microscopy and with polarized light to screen for birefringent particle deposition. Tissues from four animals from each post-exposure time point were examined. Filtered air-exposed mice were used as controls. Microscopic abnormalities were not detected in lung or nasal cavity of any animals examined, at any timepoint, suggesting that SSC exposure did not cause acute toxicity or an inflammatory response in lung or nasal cavity tissue. In addition, test article was not observed in lung or nasal cavity of exposed mice using either brightfield or polarizing light microscopy. Given that the SSC particles generated were small (1 µm diameter) and colorless, it is not unexpected that they could not be visualized with routine light microscopy and the relatively low amount of material deposited likely resulted in very few particles per histology slide to observe. Representative histopathologic micrographs are presented in Supplemental Figures 2–5.

### Hematology

A CBC assay is a valuable tool in murine toxicology studies. It evaluates hematological parameters, assessing hematotoxic effects, organ function, coagulopathy, and overall health. This comprehensive assessment helps understand the toxic potential of various substances. A small decrease in the percentage of circulating monocytes and eosinophil number was observed compared to controls at 56 days post-exposure. Migration to the lungs in response to pulmonary injury could lead to a transient decrease in monocyte and eosinophil peripheral blood count; however, the lack of observed inflammation in the lung makes this unlikely. It is possible that there may be effects of the SSC exposure on hematopoiesis or cell survival that could contribute to the observed change. The results from the CBC analysis are listed in Table 1.

### Lung inflammatory markers

**Figure 8.**
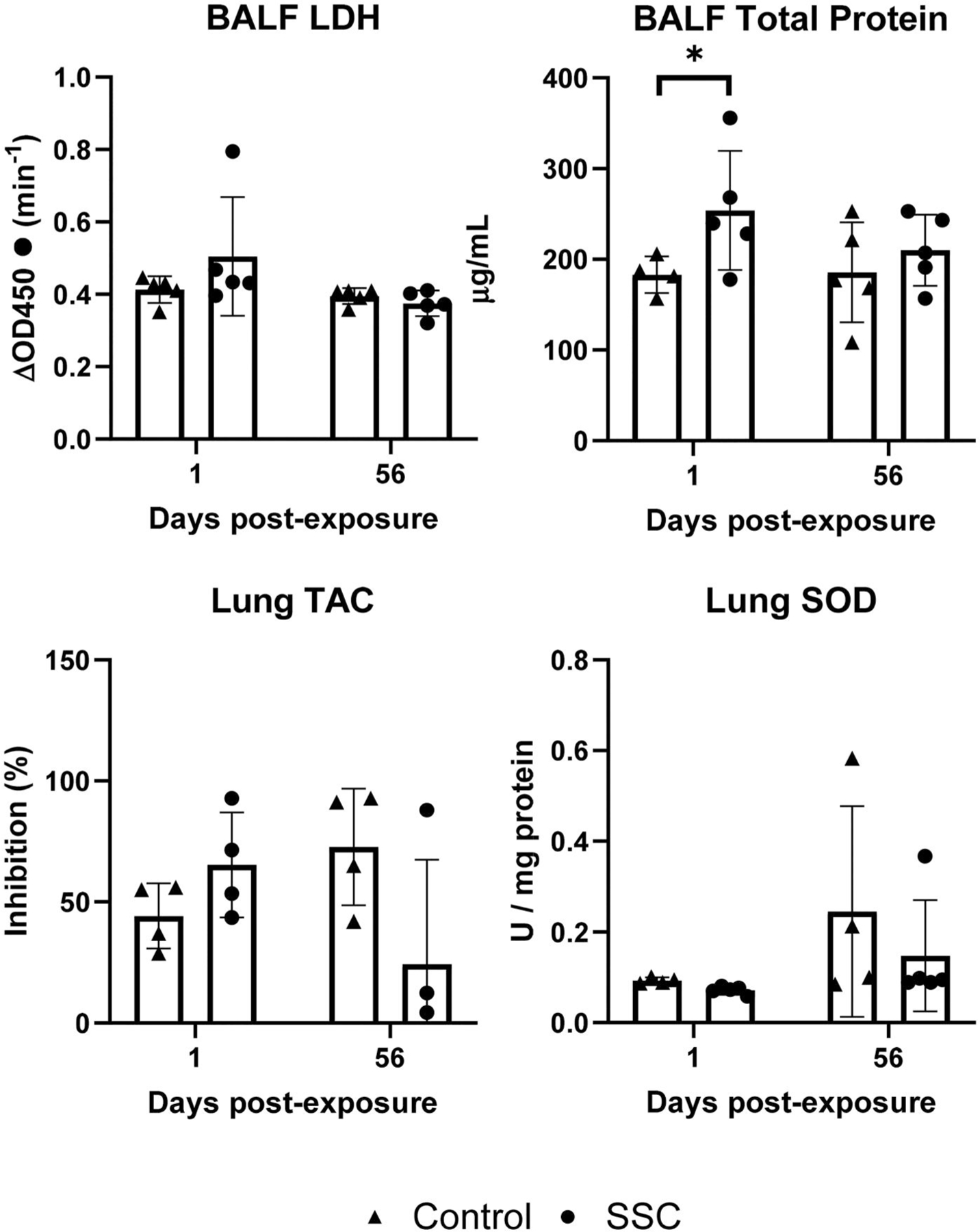
BALF inflammatory markers and lung tissue antioxidant status. No changes compared to controls were observed in BALF LDH, lung TAC, or lung SOD. BALF total protein was increased in the SSC exposed animals at 1-day post-exposure (253.9 ± 65.59 µg/mL vs 182.9 ± 17.86 µg/mL). * indicates difference from control, *p* < 0.05.

Lung tissue TAC and SOD activity are critical markers of oxidative stress, a common mechanism of pulmonary toxicity. TAC reflects the total antioxidant capacity, while SOD specifically neutralizes superoxide radicals. Elevated LDH levels in BALF indicate tissue damage, often associated with pulmonary inflammation. BALF total protein serves as a marker of alveolar-capillary barrier dysfunction, a hallmark of lung injury. No changes in lung tissue TAC, SOD activity, or BALF LDH activity were observed, however BALF total protein content was elevated compared to control in the Day 1 animals (Figure 8).

The measurement of BALF cytokines provides crucial insights into the immunological response to lung injury. IFN-γ, IL-1β, IL-6, and TNF-α are pro-inflammatory cytokines involved in initiating and amplifying the inflammatory response. IL-2, IL-4, IL-5, and IL-10 are associated with adaptive immune responses, including T-cell activation and differentiation. IL-12p70 is a key regulator of Th1 responses, while KC/GRO is a chemokine involved in neutrophil recruitment.IL-2, IL-4, IL-10, and IL-12p70 were below the assay lower limit of quantification, and no differences were no differences in any other BALF cytokine at either time point.

**Figure 9.**
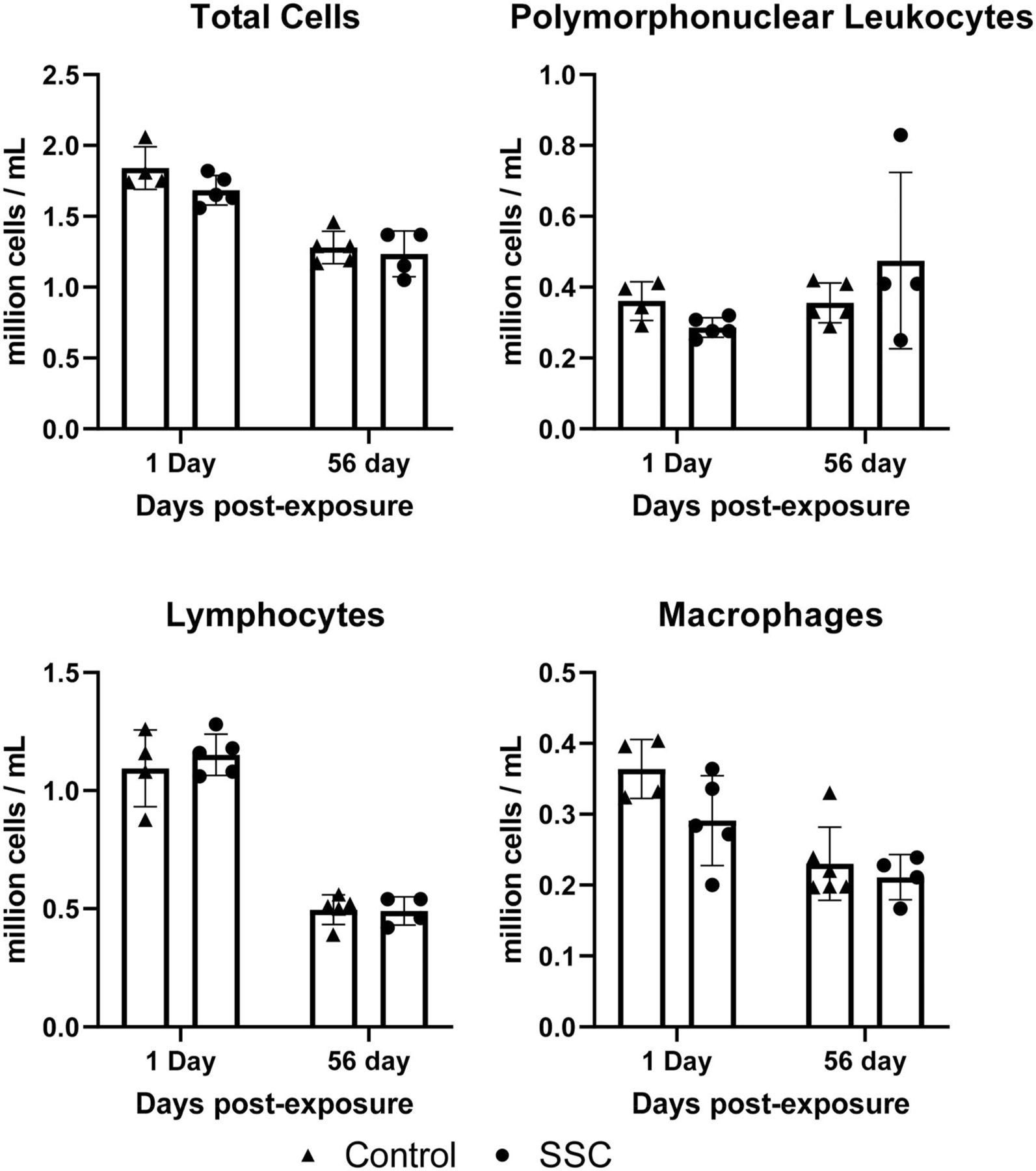
BALF cell differential. No differences at any time point were observed in BALF cell counts or cell percentages (*n* = 4–5).

Assessing total cells, macrophages, PMNs, and lymphocytes in the BLAF provides information about the pulmonary inflammatory response to particlate inhalation. Total cell counts indicate the overall extent of inflammation, while differential cell counts reveal specific immune cell populations involved. Macrophages are key players in initiating and resolving inflammation, PMNs are recruited for phagocytosis of debris and pathogens, and lymphocytes, including T cells and B cells, contribute to adaptive immune responses. No changes at any timepoint were detected in BALF cell counts (Figure 9).

## Discussion

This study sought to determine the acute SSC-induced pulmonary responses *via* whole-body inhalation exposure at 20 mg/m^3^ for 4 days, 4 h/day at 1- and 56-days post-exposure to assess the acute and sub-chronic pulmonary effects of SSC inhalation. Results showed that total estimated pulmonary deposition for these animals was 49.2 µg SSC dust/animal. No histopathologic changes were observed at any post-exposure time point; however, BALF total protein was increased at 1-day post-exposure. We conclude that exposure to dust from cutting SSC at this dose and post-exposure durations induces mild, transient inflammation.

The inherent low solubility of Al-containing dusts, including Al oxide and Al hydroxide, promotes their accumulation within the pulmonary parenchyma. This persistent presence can induce chronic inflammatory processes in the surrounding lung tissue, ultimately leading to the development of pulmonary fibrosis and impairment of the lung’s clearance function (Bomhard 2017). We observed a decrease in lung Al content measurement from 19.13 ± 5.03 µg/g immediately following the exposure period to 15.17 ± 2.26 µg/g at 24 h post exposure, an apparent clearance of 20.7%. However, there was no statistical difference between the two values, so this change may be attributed to inter-mouse and measurement deposition variability. While there are no other data regarding lung clearance of ATH available, other species of Al have been shown to be poorly cleared. Al oxide particles (mass median aerodynamic diameter (MMAD) 1.2 µm) administered *via* IT in rats was not significantly cleared at 35 weeks post exposure, although the slight negative slope in measurements over this period indicated a decrease of 9%. That study also did not observe any lung inflammation or pathology (Schlesinger et al. 2000). In rats exposed to Al oxide dust *via* inhalation (0.5–7 µm diameter) for 10 months (concentration not reported), the average lung burden was 10 mg. Lung Al remained elevated for as long as 10 months post-exposure, although it had decreased to 2.8 mg at 3 months and 0.1 mg at 6 months post-exposure (Christie et al. 1963). Considering the low clearance rate of Al oxide and the relatively short post-exposure duration of the present study, we expect that SSC clearance was minimal in all but the 56-day group.

For any novel experimental model exposure model, it is critical that the test articles are delivered in a consistent and reproducible manner. One method to evaluate the validity our apparatus is to compare it to a computational model system. The Multiple-Path Particle Dosimetry Model (MPPD v3.4, Applied Research Associates, Albuquerque, NM) is a software tool used to estimate the deposition and clearance of aerosols in humans and laboratory animals. For a spherical particle with a density of 1.7 g/cm^3^(DuPont 2015), with a CMAD of 820 nm, the MPPD software predicted a total lung dose of 12 µg/mouse for a 4-h exposure at 20 mg/cm^3^ in a 20 g mouse. This estimate closely matched the observed 13.2 µg/mouse per day that was achieved in the experimental animals, suggesting that our system can generate and deliver aerosols that are representative of real time inhalation exposure.

Our target exposure concentration of 20 mg/m^3^ was arrived at *via* the input of several factors. First, in testing, the chamber could generate and maintain up to about 40 mg/m^3^, but lower concentrations were less variable. Second, Vinnikov et al. reported workday (8.2 ± 0.5 h) average concentrations of 2.66 ± 2.31 mg/m^3^, with peaks as high as 25.4 mg/m^3^ (Vinnikov et al. 2021). We chose 20 mg/m^3^ (10 mg/m^3^, 8 h – TWA) to represent a high but still occupationally relevant level of exposure. To translate murine model dosages to human exposure time, several parameters were extrapolated. An occupational scenario of inhaling 50% of the OSHA PEL for PNOR(i.e. 2.5 mg/m^3^), composed of SSC aerosol with a measured MMAD of 1.79 lm, was assumed. This results in an estimated 10% alveolar deposition fraction (Phalen 1984). Additionally, a minute ventilation rate of 0.02 m^3^/min for light work (Galer et al. 1992) and an average human alveolar epithelial surface area of 102 m^2^ (Stone et al. 1992) were considered. Finally, a typical exposure scenario of 4-h shifts, 5 days/week, for 50 weeks/year was employed. Using these parameters, we can calculate the estimated human equivalent exposure years using equations using Equations (1)–(4) in the supplement. Applying these equations, we found that the lung deposition achieved in the present inhalation study of 49.2 µg, in the absence of clearance, is equivalent to a human working for 122 days at an average concentration of 2.5 mg/m^3^ or 152 days at the average (2 mg/m^3^) occupational concentration observed by Vinnikov et al.

Our previous *in vivo* study using SSC material indicated a moderate potential for pulmonary toxicity(Mandler et al. 2019). In that study, mice were exposed to a range of SSC doses from between 62.5 and 1000 µg per animal using oropharyngeal aspiration and examined 1 day and 14 days post-exposure. Oropharyngeal aspiration is widely recognized as a valid analogue for inhalation (Porter et al. 2010; Kinaret et al. 2017), resulting in distribution into the deep lung. The lowest aspirated dose of 62.5 µg was higher than the inhaled deposition of 49.2 µg over 4 days of exposure achieved in the present study; however, there may still be some valid comparisons drawn between the two outcomes. In the earlier study, the geometric mean particle diameter as dispersed in biocompatible medium was 1.16 µm, which can be compared to estimated aerodynamic diameter(d_a_) using supplemental equations 5 and 6 (Hinds 1999). Compared to a MMAD of 1.79 µm for the present study, the particles used in the previous *in vivo* aspiration study appear to have been somewhat smaller (d_e_ = 1.56 µ*m*). Our observations of particle size were much closer to that observed by Qi et al. (1.78 µm) using a similar automated cutting apparatus and measured *via* MOUDI. The differences in particle size distribution between these studies may be attributed to differences in measurement method (calculated aerodynamic diameter vs MOUDI) or that suspension in dispersion medium necessary for oropharyngeal aspiration may some-what alter the size distribution compared to real time-generated particles (i.e. biocorona formation or agglomeration). It is possible that some of the differences in observed effects in the lung between the two exposures may owe to the somewhat larger particle size in the present study, as smaller particles generally produce a greater biologic effect on a per mass basis (Schraufnagel 2020), although the relatively small difference likely is sufficient to be physiologically relevant.

In the earlier SSC aspiration study, at the lowest 62.5 µg dose, we observed moderate acute bronchiolitis and alveolitis at 24-h post exposure. Additionally, neutrophilic infiltrates were observed at terminal bronchioles with extension into alveolar ducts and adjacent alveoli, with dose-dependent increases in inflammatory markers as the exposure bolus increased. The observed mild inflammation in the 62.5 µg was resolved in the 14-day animals. In contrast, we did not observe any signs of alveolitis or cellular infiltrates following SSC inhalation at any time point. This was likely due to the lower exposure level achieved *via* inhalation in this study and that the exposure occurred over multiple days, rather than in a single bolus. A transient decrease in alveolar microvascular integrity may explain our observed increase in BALF total protein. Future studies should use repeated exposures to achieve higher lung burdens. In this study, there were no significant changes in pulmonary inflammation between the 1- day and 56-day post-exposure groups. However, it is likely that a transient intermediate increase in pulmonary inflammation between day 1 and day 56 post-exposure may have been missed. Additional post-exposure time points may be needed in future studies to detect the dynamic changes in pulmonary inflammation following SSC exposure.

This study only investigated a single exposure dose, representing a relatively short equivalent worker exposure and post exposure timepoints that focused on development and resolution of active inflammation. Confirmation of these findings would necessitate testing additional, potentially higher, lung depositions incurred over longer exposure periods. We also only generated dust using a single method (sawing). While there are no studies specifically investigating the effects of tool selection and engineering controls on SSC aerosol generation, they can have substantial impacts on aerosol generation during engineered stone countertop fabrication (Johnson et al. 2017), and it is likely that similar patterns are present during SSC fabrication. Finally, it is important to note that the study solely employed one type of solid surface composite, thus limiting the generalizability of results to exposures from other materials.

## Conclusions

We designed a particle generation and inhalation exposure system for solid surface composite materials. The particles generated were similar in size distribution to those generated in workplace and laboratory environments, resulting in measured lung deposition values that closely matched those predicted by modeling software. Future studies should endeavor to reach greater lung depositions that reflect longer-term workplace exposures. This exposure system will prove valuable for further investigations of emissions from working with SSC and other novel construction materials.

## Supplementary Material

Supplementary Material

## Figures and Tables

**Table 1.** Hematological parameters.

	Day 1 Air	Day 1 SSC	Day 56 Air	Day 56 SSC
RBC	7.07±1.55	7.82±1.96	9.45±0.396	9.11±0.946
HGB	10.7±2.19	11.7±2.94	13.6±0.63	13.2±1.28
HCT	34.0±8.04	38.5±10.6	44.8±2.01	43.1±4.68
MCV	47.9±0.86	48.9±1.75	47.4±0.81	47.3±0.67
MCH	15.2±0.27	15.0±0.23	14.3±0.24	14.5±0.16
MCHC	31.8±1.01	30.7±1.10	30.3±0.36	30.7±0.46
RDW-SD	26.5±2.04	26.9±1.54	27.9±1.06	27.7±1.17
RDW-CV	19.3±2.75	20.4±2.52	23.7±0.87	23.2±1.30
RET	280.1±102.9	320.0±121.4	376.8±47.61	355.2±51.12
RET %	3.86±0.812	4.07±1.03	3.98±0.45	3.89±0.298
WBC	1.13±1.17	1.43±0.75	3.59±1.69	2.93±0.77
NEUT	0.19±0.29	0.25±0.23	0.42±0.24	0.33±0.10
NEUT %	15.6±10.5	17.6±13.7	11.7±4.5	11.5±3.04
LYMPH	0.887±0.851	1.09±0.618	2.98±1.46	2.49±0.707
LYMPH %	79.3±8.15	76.6±12.5	82.4±6.56	84.6±3.39
MONO	0.023±0.018	0.020±0.027	0.050±0.015	0.037±0.016
MONO %	2.14±1.54	1.29±1.63	1.58±0.76	*1.34±0.64
EO	0.03±0.03	0.06±0.04	0.14±0.08	*0.07±0.03
EO %	2.93±1.92	4.24±1.67	4.19±2.10	2.54±0.467
BASO	0.001±0.004	0.006±0.007	0.003±0.005	0.002±0.004
BASO %	0.04±0.11	0.34±0.49	0.10±0.21	0.06±0.11

* indicates *p<*0.05 versus time-matched air control group (*n*=9). Definitions for each parameter can be found in S9.

## Data Availability

The data that support the findings of this study are available from the corresponding author, WKM, upon reasonable request. The findings and conclusions in this report are those of the authors and do not necessarily represent the official position of the National Institute for Occupational Safety and Health, Centers for Disease Control and Prevention.
