# Supplementary material for: Mouse pulmonary response following solid surface composite dust inhalation
Source: Inhal Toxicol. Author manuscript; Available in PMC 2025 May 14. (PMC12077238; doi:10.1080/08958378.2024.2447699)
Supplement: Supplementary Material [file NIHMS2060765-supplement-Supplementary_Material.docx]

**Supplemental Figures**


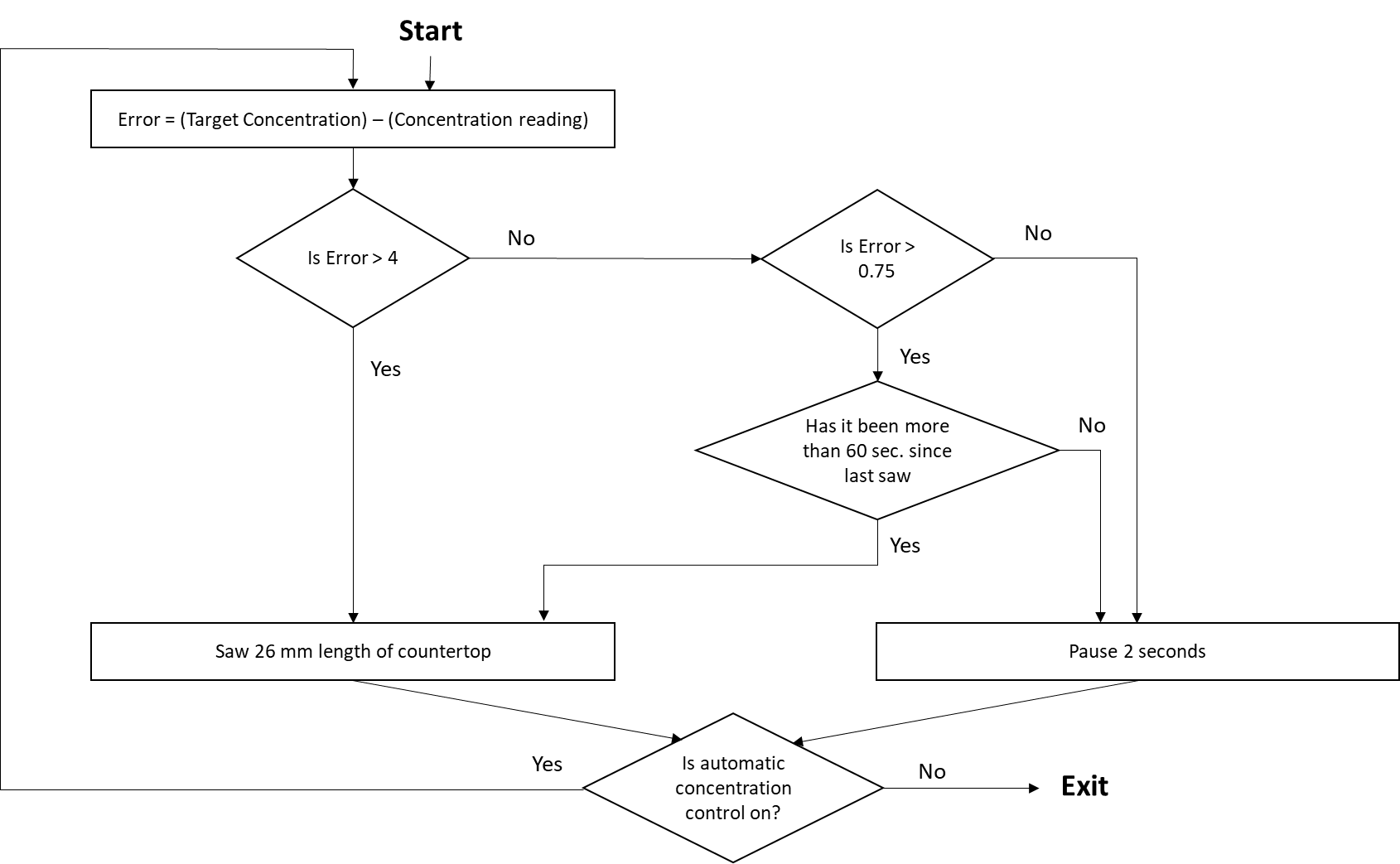


S1. Concentration control algorithm flow chart. Graphical representation of the algorithm used by the particle generation apparatus to maintain chamber particle concentration via activation of saw and step motor.


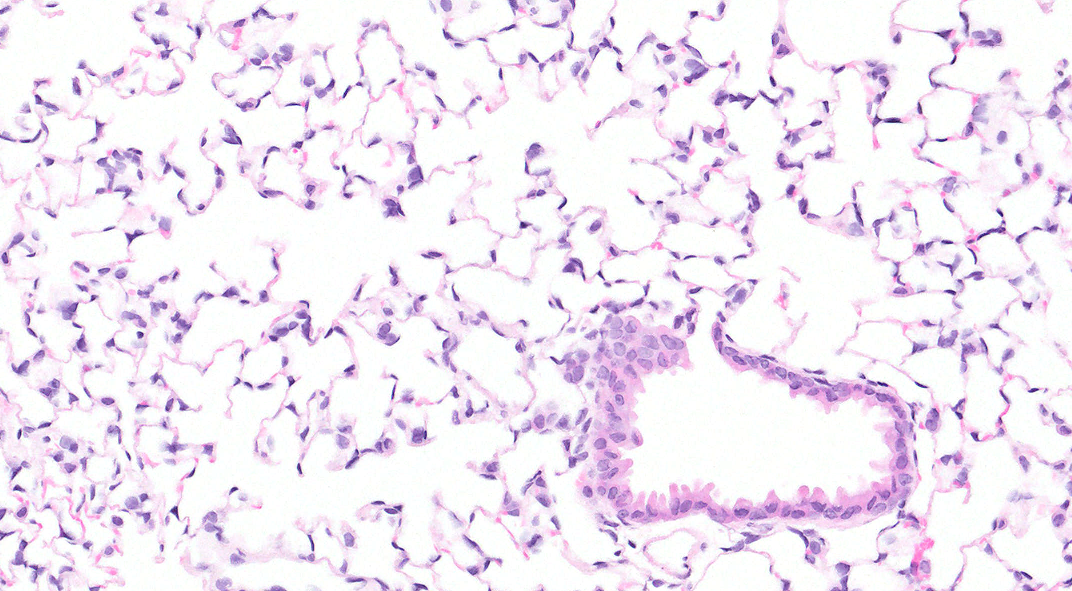


S2. Representative micrograph of mouse lung exposed to filtered air control at 1-day post-exposure, stained in H&E. 20X magnification.


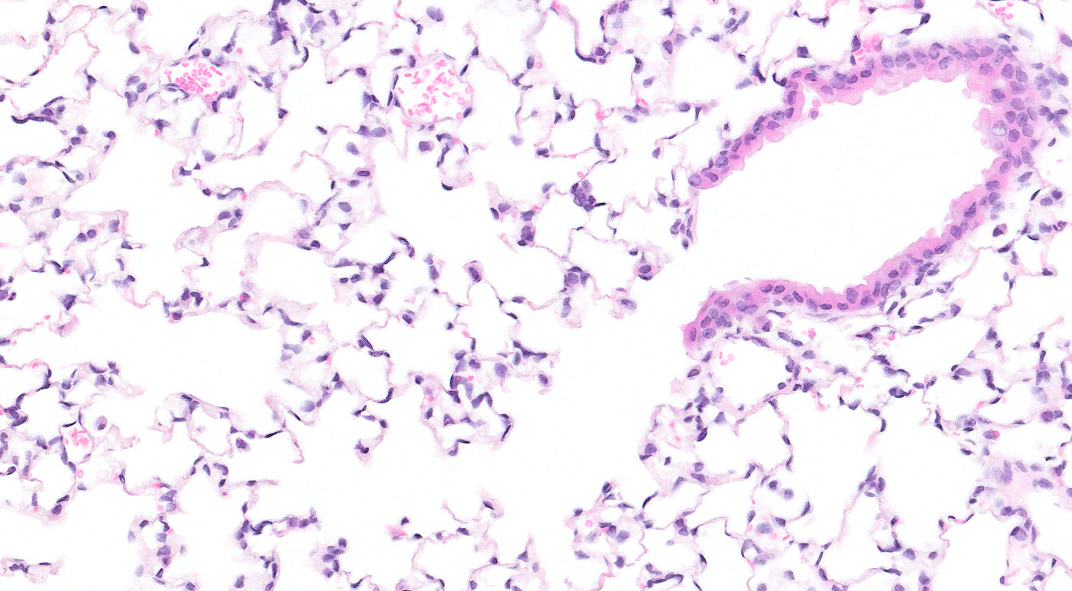
S3. Representative micrograph of mouse lung exposed to filtered air control at 56 days post-exposure, stained in H&E. 20X magnification.


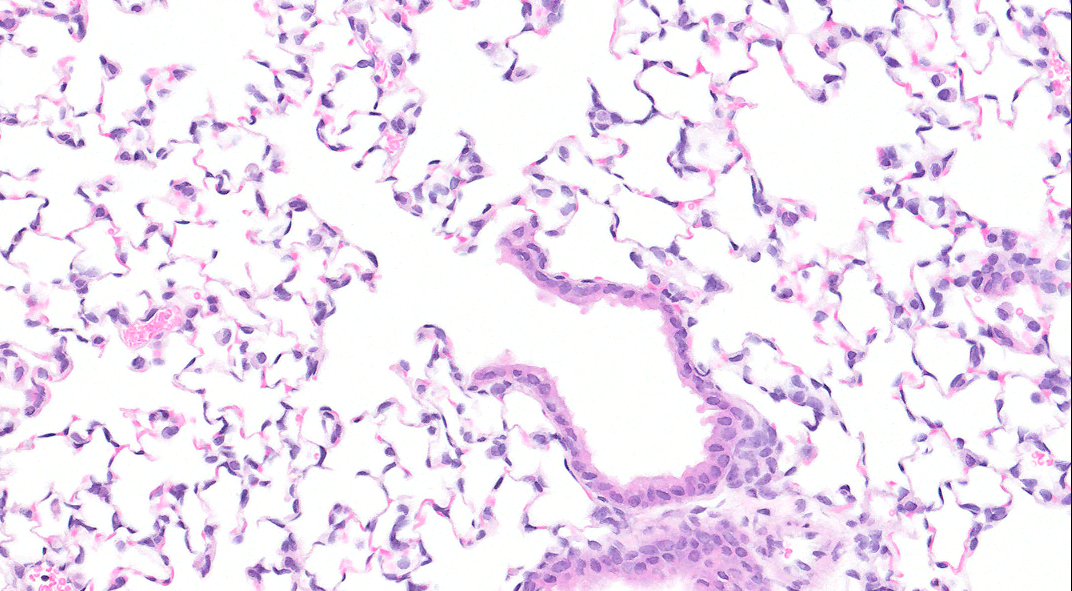


S4. Representative micrograph of mouse lung exposed to emissions from cutting SSC for 4 days, 4 hours / day, at 1-day post-exposure, stained in H&E. 20X magnification.


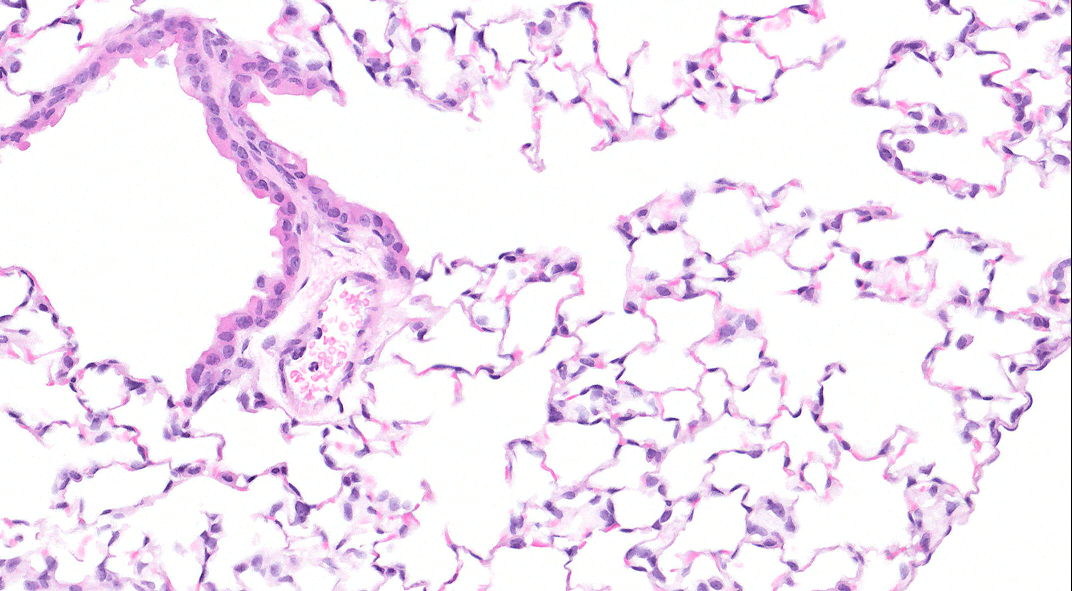
S5. Representative micrograph of mouse lung exposed to emissions from cutting SSC for 4 days, 4 hours / day, at 56 days post-exposure, stained in H&E. 20X magnification.


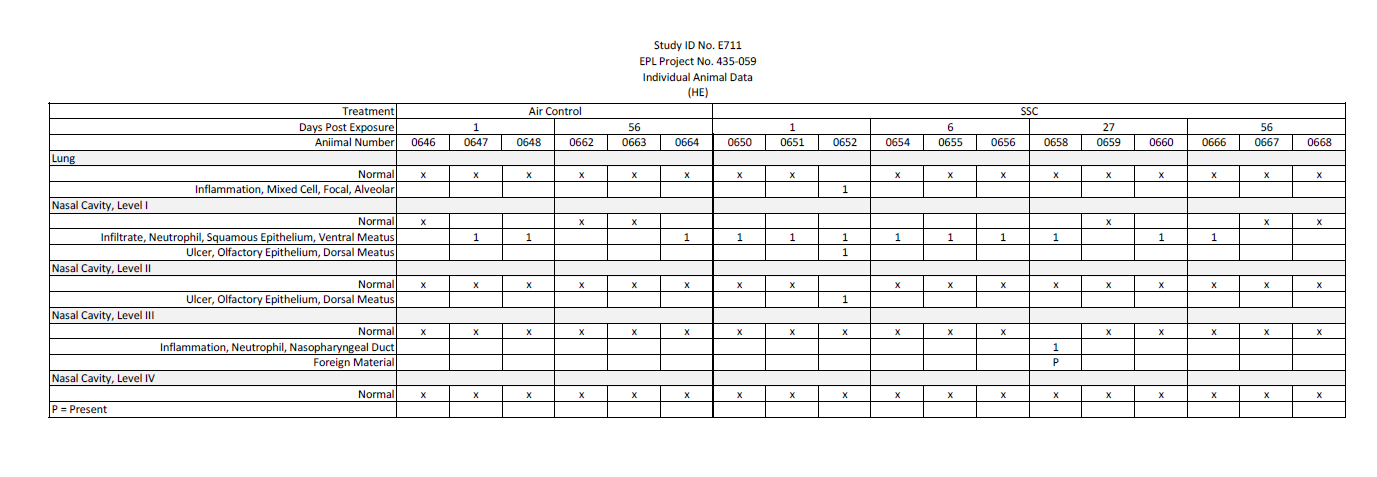
S6. Histology scores for mouse lungs and nasal sections I-IV exposed to exposed to emissions from cutting SSC or filtered air for 4 days, 4 hours / day.

S7. Data Dictionary

| **Field Term** | **Definition** |
| --- | --- |
| RBC(M/µL) | Erythrocytes - functional component of blood responsible for the transportation of gases |
| HGB(g/dL) | Hemoglobin - protein in red blood cells that carries oxygen. |
| HCT(%) | Hematocrit - the percentage by volume of red cells |
| MCV(fL) | Mean corpuscular volume - measurement of the average size of red blood cells |
| MCH(pg) | Mean corpuscular hemoglobin -average amount of hemoglobin found in the red blood cells |
| MCHC(g/dL) | Mean Corpuscular Hemoglobin Concentration - measurement of the amount of hemoglobin a red blood cell has relative to the cell's volume |
| RDW-SD(fL) | Red Cell Distribution Width - heterogeneity of cell size in the peripheral blood |
| RDW-CV(%) | Red Cell Distribution Width deviation |
| RET# (K/µL) | Reticulocytes - immature red blood cells (RBCs) produced in the bone marrow and released into the peripheral blood |
| RET (%) | % Reticulocytes |
| PLT (K/µL) | Platelets - cell fragments that form clots and stop or prevent bleeding |
| PDW (fL) | Platelet Distribution Width - measurement of platelet anisocytosis calculated from the distribution of individual platelet volumes |
| MPV (fL) | Mean Platelet Volume |
| P-LCR (%) | Platelet-large cell ratio - percentage of all platelets with a volume measuring over 12 fL circulating in the bloodstream |
| PCT (%) | Plateletcrit - volume occupied by platelets in the blood |
| WBC (K/µL) | White Blood Cells - granulocytes (neutrophils, eosinophils, and basophils) and non-granulocytes (lymphocytes and monocytes) |
| NEUT #(K/µL) | Neutrophils - most abundant type of granulocytes |
| LYMPH #(K/µL) | Lymphocytes - type of white blood cell that is part of the immune system |
| MONO #(K/µL) | Monocytes - white blood cells that derive from the bone marrow |
| EO# (K/µL) | Eosinophils - granulocytes that express cytoplasmic granules that contain basic proteins and bind with acidic dyes |
| BAS# (K/µL) | Basophils - least common type of granulocyte |
| NEUT (%) | Neutrophils percent |
| LYMPH (%) | Lymphocytes percent |
| MONO (%) | Monocytes percent |
| EO (%) | Eosinophils percent |
| BASO (%) | Basophils percent |
| LDH (U/L) | Lactate dehydrogenase - LDH is an enzyme that is found in all cells, but it is released into the bloodstream when cells are damaged. A high LDH level in BALF can indicate that there is damage to the lung tissue |
| IFN-y (pg/mL) | Interferon gamma - cytokine involved in innate and adaptive immunity |
| IL-10 (pg/mL) | Interleukin-10 - cytokine with effects in immunoregulation and inflammation |
| IL-13 (pg/mL) | Interleukin-13 - cytokine that promotes the differentiation of Th2 cells and the production of IgE antibodies |
| IL1-β (pg/mL) | Interleukin 1 beta - proinflammatory cytokine that promotes inflammation, fever, cytokine production, T cell activation, and bone resorption |
| IL-4 (pg/mL) | Interleukin-4 - cytokine that promotes Th2 cell differentiation and is involved in allergic inflammation. |
| IL-5 (pg/mL) | Interleukin -5 - cytokine that promotes the differentiation and activation of eosinophils |
| IL-6 (pg/mL) | Interleukin-6 - pleiotropic cytokine that regulates inflammation, immunity, and metabolism |
| KC/GRO (pg/mL) | Keratinocyte chemoattractant /human growth-regulated oncogene - measures the levels of two proteins, keratinocyte chemoattractant and human growth-regulated oncogene, which are associated with the development of cancer |
| TNF-α (pg/mL) | Tumor necrosis factor - alpha - proinflammatory cytokine that plays a role in a variety of cellular processes, including cell proliferation, differentiation, and apoptosis |
| TP (mg/mL) | Total protein - amount of protein in lung fluid. High levels can indicate conditions like pulmonary edema, pneumonia, fibrosis, sarcoidosis, or lung cancer. |
| TAC - Total Antioxidant Capacity (% inhibition)- | Biomarker in quantifying the inherent antioxidant defense system within cells. |
| SOD - superoxide dismutase (U / mg protein) | Quantifies the enzymatic activity of SODs, a family of antioxidant enzymes constituting the first line of defense against oxidative stress. |

S8. Limits of detection and quantification for the V-PLEX Proinflammatory Panel.

| **Analyte** | **LLOD** | **LLOQ** | **ULOQ** | **Dynamic Range** | **Unit** |
| --- | --- | --- | --- | --- | --- |
| IFN-γ | 0.04 | 0.39 | 570 | 0.04 - 570 | pg/mL |
| IL-1β | 0.11 | 0.72 | 1,030 | 0.11 - 1,030 | pg/mL |
| IL-2 | 0.22 | 1.03 | 1,570 | 0.22 - 1,570 | pg/mL |
| IL-4 | 0.11 | 0.818 | 1,060 | 0.11 - 1,060 | pg/mL |
| IL-5 | 0.06 | 0.302 | 590 | 0.06 - 590 | pg/mL |
| IL-6 | 0.61 | 7.61 | 3,140 | 0.61 - 3,140 | pg/mL |
| IL-10 | 0.94 | 7.26 | 2,030 | 0.94 - 2,030 | pg/mL |
| IL-12p70 | 9.95 | 179 | 20,600 | 9.95 - 20,600 | pg/mL |
| KC/GRO | 0.24 | 3.29 | 1,230 | 0.24 - 1,230 | pg/mL |
| TNF-α | 0.13 | 0.98 | 403 | 0.13 - 403 | pg/mL |

S9. MPPD model parameters used to estimate mouse lung burden.

 MPPD v3.04 © 2016 by Applied Research Associates, Inc.

  --> Lung morphometry <--

  Lung Geometry: mouse

  Number of segments: 22

  Scaling tree by (TLC ---> FRC): 0.597

  TLC = 1.03 ml

  FRC = 0.22 ml

  Scaling tree by ((1+TV/2FRC) ^1/3): 1.779

  Calculated FRC =       0.22 ml

  Lung (distal) volume = 1.23 ml

  Volume of conducting airways: 0.09 ml

  -----> Breathing Parameters and Times <-------

  Breathing Frequency: 250.0 #/min     Tidal volume: 2.0268848624439206 ml

  Nasopharyngeal dead space: 0.0322 ml

  --> Regional Deposition <--

  Inhalation time: 0.12 sec    Exhalation time: 0.12 sec

  Volumetric inhalation flow rate at trachea: 16.89070718703267 ml/sec

  Volumetric exhalation flow rate at trachea: 16.89070718703267 ml/sec

  Time spent in the head during inhalation: 0.002 sec

  Time spent in the head during exhalation: 0.002 sec

  --> Particle properties <--

  Diameter: 1.790E+0 µm

  Sigma_g (GSD): 2.750E+0

  Mass density: 1.0 g/cm^3

  Aerosol concentration: 20.0 mg/m^3

  Inhalable fraction: 1.0000

  Breathing route: nasal

  --> Inhalation <--

  Inspiratory Fraction: 0.5

  --> Pause <--

  Pause Fraction: 0.0

  Total head deposition fraction: 0.9252

  Total TB deposition fraction: 0.0145

  Total pulmonary deposition fraction: 0.0122

  Total deposition fraction: 0.9519


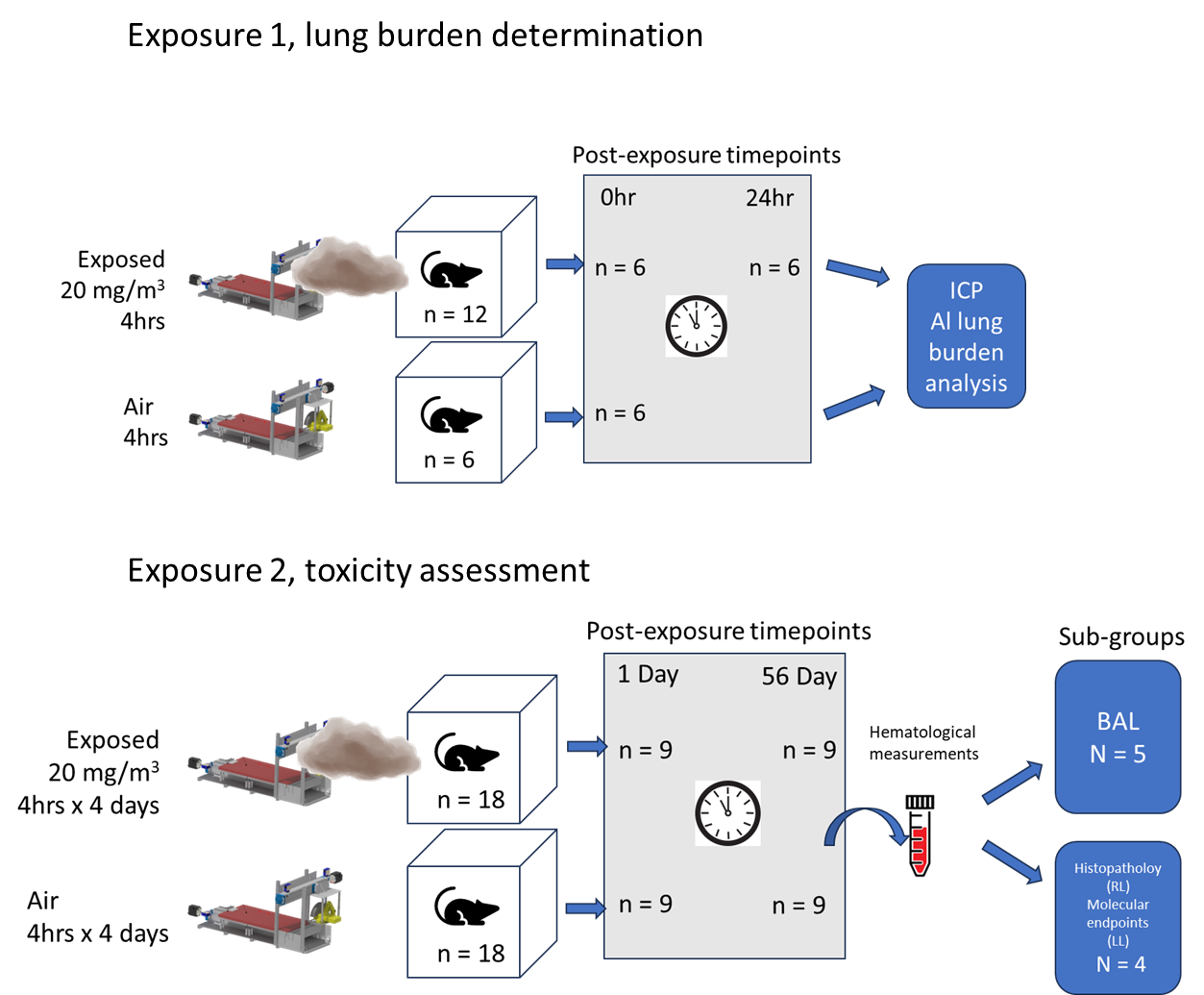


S10. Experimental design diagram.

S11. Histopathology scoring system

**Grade 1 (1+): Minimal.** This corresponds to a histopathologic change ranging from

inconspicuous to barely noticeable but so minor, small, or infrequent as to warrant no more than

the least assignable grade. For multifocal or diffusely-distributed lesions, this grade was used

for processes where less than approximately 10% of the tissue in an average high-power field

was involved. For focal or diffuse hyperplastic/hypoplastic/ atrophic lesions, this grade was

used when the affected structure or tissue had undergone a less than approximately 10%

increase or decrease in volume.

**Grade 2 (2+): Mild.** This corresponds to a histopathologic change that is a noticeable but not a

prominent feature of the tissue. For multifocal or diffusely-distributed lesions, this grade was

used for processes where between approximately 10% and 25% of the tissue in an average

high-power field was involved. For focal or diffuse hyperplastic/hypoplastic/atrophic lesions, this

grade was used when the affected structure or tissue had undergone between an approximately

10% to 25% increase or decrease in volume.

**Grade 3 (3+): Moderate.** This corresponds to a histopathologic change that is a prominent but

not a dominant feature of the tissue. For multifocal or diffusely-distributed lesions, this grade

was used for processes where between approximately 25% and 50% of the tissue in an

average high-power field was involved. For focal or diffuse hyperplastic/hypoplastic/atrophic

lesions, this grade was used when the affected structure or tissue had undergone between an

approximately 25% to 50% increase or decrease in volume.

**Grade 4 (4+): Marked.** This corresponds to a histopathologic change that is a dominant but not

an overwhelming feature of the tissue. For multifocal or diffusely-distributed lesions, this grade

was used for processes where between approximately 50% and 95% of the tissue in an

average high-power field was involved. For focal or diffuse hyperplastic/hypoplastic/atrophic

lesions, this grade was used when the affected structure or tissue had undergone between an

approximately 50% to 95% increase or decrease in volume.

**Grade 5 (5+): Severe.** This corresponds to a histopathologic change that is an overwhelming

feature of the tissue. For multifocal or diffusely-distributed lesions, this grade was used for

processes where greater than approximately 95% of the tissue in an average high-power field

was involved. For focal or diffuse hyperplastic/hypoplastic/atrophic lesions, this grade was used

when the affected structure or tissue had undergone a greater than approximately 95% increase

of decrease in volume.

Supplemental Equations

*Eq. 1. Exposure rate = Minute ventilation * Permissible Exposure Limit * Deposition fraction*

$$\frac{0.005 mg}{min}= \frac{0.02m^{3}}{\text{min }}* \frac{2.5 mg}{m^{3}}* 10\%$$

*Eq. 2. Yearly deposition = Exposure rate * Exposure duration*

$\frac{300 mg}{year\text{ }}$ = $\frac{0.005 mg}{min} *\frac{60 min}{hour}*\frac{4 hrs}{day}*\frac{5 days}{week}*\frac{50 weeks}{year}$

*Eq. 3. Exposure relative to alveolar surface area = Yearly deposition / human alveolar surface area*

$$\frac{2.94 mg/m^{2}}{\mathrm{year}}= \frac{300 mg}{102m^{2}}$$

*Eq. 4. Mouse alveolar surface area / exposure relative to alveolar surface area = Human equivalent exposure years*

$\frac{Experimental Deposition (mg)}{0.05m^{2}}$ / $\frac{2.94 mg/m^{2}}{\mathrm{year}}$ = *Human equivalent exposure years*

First, the equivalent volume diameter (d_e_) must be calculated using Eq .5 using the measured geometric diameter (d_PA_) with a volume shape factor (α) of .21 (Hinds 1999).

*Eq. 5. Particle equivalent volume diameter*

$$de=d_{PA}\left( \frac{6\alpha_{v}}{\pi} \right)^{\frac{1}{3}}$$

$$de=1.16\mu m\left( \frac{6*0.21}{\pi} \right)^{1/3}=0.85\mu m$$

*Eq. 6. Particle aerodynamic diameter*

$$d_{a}=d_{e}\left( \frac{\rho_{p}}{\rho_{0}x} \right)^{1/2}$$

$$d_{a}=1.16\mu m\left( \frac{1800\frac{\mathrm{kg}}{m^{3}}}{1000\frac{\mathrm{kg}}{m^{3}}*0.839} \right)^{1/2}=1.56 \mu m$$

With d_e_ determined, the aerodynamic diameter may be calculated (d­_­a_) using Eq. 6, where Ρρ is the particle density (1800 kg/m^3^) (DuPont 2015, 2016), Ρ_0_ is the standard particle density of 1000 kg/m^3^, and X is the dynamic shape factor of 0.839 (Hsieh 1985).
